# Adult presentation of Simpson-Golabi-Behmel syndrome due to a hemizygous *GPC3* stopgain variant mimicking acromegaly

**DOI:** 10.1210/jcemcr/luag115

**Published:** 2026-05-28

**Authors:** R K Rishabh, Satyavamsi Gadde, Himamshu Acharya, Vivekananda Bhat, Kishan Delampady, Ganesh HK

**Affiliations:** Department of Endocrinology, AJ Institute of Medical Sciences, Mangaluru, Karnataka 575004, India; Department of Endocrinology, AJ Institute of Medical Sciences, Mangaluru, Karnataka 575004, India; Department of Endocrinology, AJ Institute of Medical Sciences, Mangaluru, Karnataka 575004, India; Department of Medical Genetics, Kasturba Medical College, Manipal Academy of Higher Education, Manipal, Karnataka 576104, India; Department of Endocrinology, NMC Royal Hospital, Sharjah 70667, United Arab Emirates; Department of Endocrinology, AJ Institute of Medical Sciences, Mangaluru, Karnataka 575004, India

**Keywords:** acromegaly, Simpson-Golabi-Behmel syndrome, apparent acromegaly, overgrowth syndromes, accessory nipples, supernumerary nipples

## Abstract

Simpson-Golabi-Behmel syndrome (SGBS) is a rare, X-linked overgrowth disorder caused by pathogenic variants in the *GPC3* gene, which encodes glypican-3. It is characterized by prenatal and postnatal overgrowth, craniofacial dysmorphism, supernumerary nipples, skeletal anomalies, and variable neurodevelopmental delay. Because of overlapping features such as macroglossia and coarse facial appearance, SGBS may be mistaken for acromegaly. We report a 33-year-old man evaluated for apparent acromegaloid facies. He had tall stature (196.5 cm; >97th percentile), macroglossia, mandibular prognathism, hypertelorism, supernumerary nipples, delayed developmental milestones, and mild intellectual disability. His brother had similar but milder features. Serum insulin-like growth factor 1 concentration was 75.9 ng/mL (SI: 9.95 nmol/L) (reference range, 94-284 ng/mL [SI: 12.3-37.2 nmol/L]), and growth hormone suppressed to 0.49 ng/mL (SI: 0.49 µg/L) following an oral glucose tolerance test, excluding acromegaly. Magnetic resonance imaging showed a 2.2-mm pituitary microadenoma considered incidental. Exome sequencing identified a hemizygous nonsense pathogenic variant in *GPC3* (c.1159C > T; p.Arg387Ter), confirming SGBS type 1; the mother was heterozygous. This case highlights that tall stature with dysmorphic features, and supernumerary nipples, should prompt consideration of SGBS, with endocrine testing used to exclude acromegaly.

## Introduction

Acromegaly is a disorder of pathological somatic overgrowth caused by chronic excess growth hormone (GH), most commonly due to a GH-secreting pituitary adenoma. Diagnosis is established by elevated insulin-like growth factor 1 (IGF-1) levels and failure of GH suppression during an oral glucose tolerance test (OGTT), with pituitary imaging used to identify adenomas [1].

However, not all individuals with acromegaloid features have acromegaly. Several genetic overgrowth syndromes can mimic acromegaly due to macroglossia, coarse facial features, and tall stature. These include X-linked acrogigantism (X-LAG), Sotos syndrome, Weaver syndrome, Beckwith-Wiedemann syndrome, and Simpson-Golabi-Behmel syndrome (SGBS) [2].

SGBS is a rare, X-linked overgrowth disorder caused by disease-causing single nucleotide variants in *GPC3*, located at Xq26.2. It is characterized by prenatal and postnatal overgrowth, distinctive craniofacial features, supernumerary nipples, skeletal anomalies, variable intellectual disability, and increased tumor risk [3]. Recognition of syndromic features is critical for early diagnosis and appropriate surveillance.

## Case presentation

A 33-year-old man was admitted for evaluation of breathlessness and cough. He had a history of pulmonary tuberculosis and later developed post–tubercular bronchiectasis. An endocrinology evaluation was sought for acrogigantic features.

On examination, he was noted to have striking acromegaloid facies. His height was 196.5 cm (>97th percentile), weight 88.5 kg, and body mass index 22.9. Arm span was 198 cm. Head circumference was 59 cm (>97th percentile). Family members reported that he had always been taller than peers from early childhood.

He was born at home and delivered by a midwife. Formal birth records, including birth weight and length, were unavailable. According to maternal recall, he was described as larger than average at birth, although precise measurements could not be confirmed. There was no documented history of neonatal intensive care admission.

Developmental history revealed delayed speech and motor milestones. He had mild intellectual disability and completed limited formal education.

**Figure 1:**
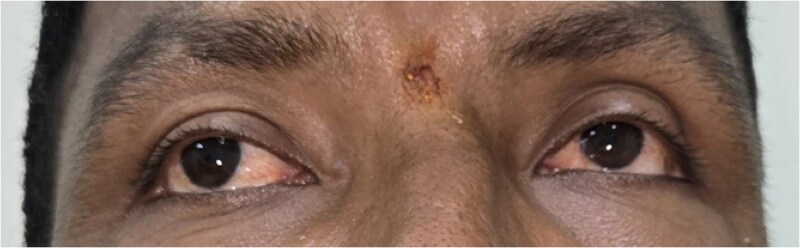
Clinical photograph showing hypertelorism with a broad nasal bridge.

**Figure 2:**
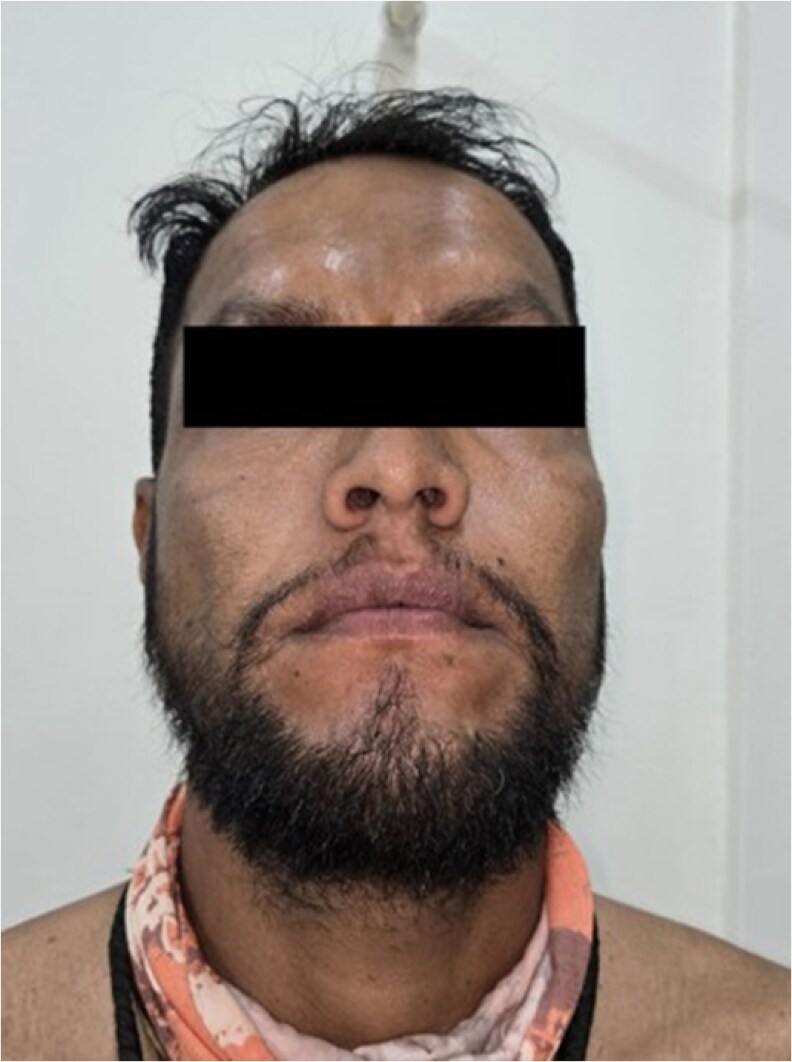
Frontal view of the face demonstrating coarse acromegaloid facies with mandibular prognathism and thickened lips.

**Figure 3:**
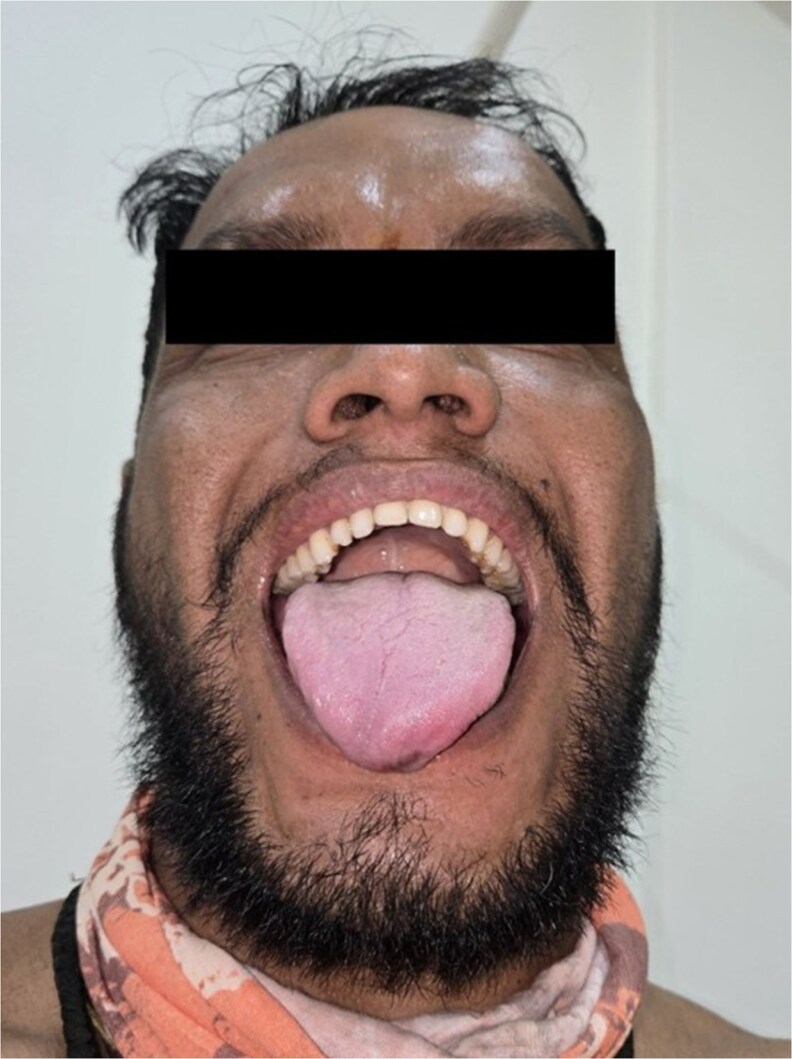
Clinical photograph showing macroglossia with prominent tongue protrusion and partially widened dental spacing.

**Figure 4:**
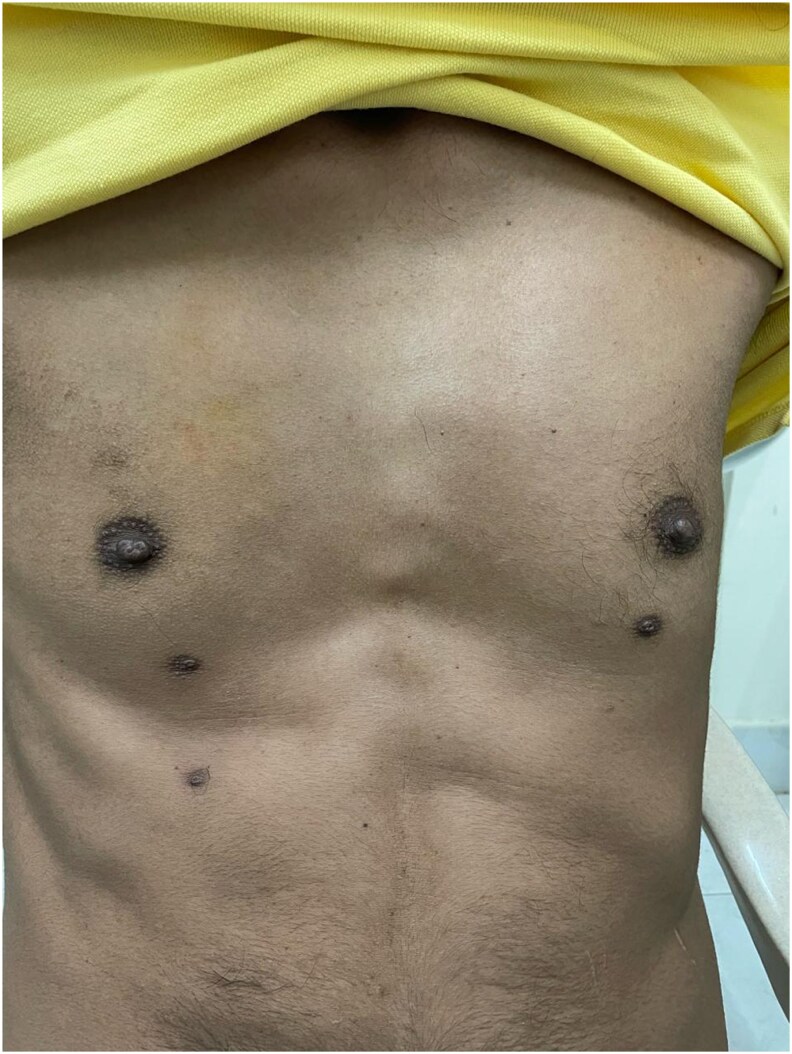
Chest examination revealing supernumerary nipples in addition to normally positioned nipples.

Dysmorphic features included hypertelorism, broad nasal bridge (Fig. 1), tubular nose, short philtrum, macrostomia, thick lips, mandibular prognathism, macroglossia (Figs. 2 and 3), broad hands with short distal phalanges, hypoplastic nails, genu valgum, lateral deviation of toes, and bilateral supernumerary nipples (Figs. 4).

There were hypopigmented macules over the trunk. No gynecomastia was noted.

Cardiovascular examination was unremarkable. Electrocardiography demonstrated normal sinus rhythm without conduction abnormalities or arrhythmias.

Family history revealed a younger brother (height 192 cm) with similar but milder facial features. The brother had a normal intellect and no supernumerary nipples. The maternal uncle had a tall stature (no anthropometric data available). Apart from the 2 siblings and their maternal uncle, no other family member had such history. The mother (height 168 cm) had no overt dysmorphic features.

## Diagnostic assessment

Given the acromegaloid appearance, endocrine evaluation was performed to exclude acromegaly. The serum IGF-1 concentration was 75.9 ng/mL (SI: 9.95 nmol/L) (reference range, 94-284 ng/mL [SI: 12.3-37.2 nmol/L]). GH was suppressed to 0.49 ng/mL (SI: 0.49 µg/L) following OGTT (<1.0 ng/mL or <1 µg/L considered normal). These findings excluded GH excess.

**Figure 5:**
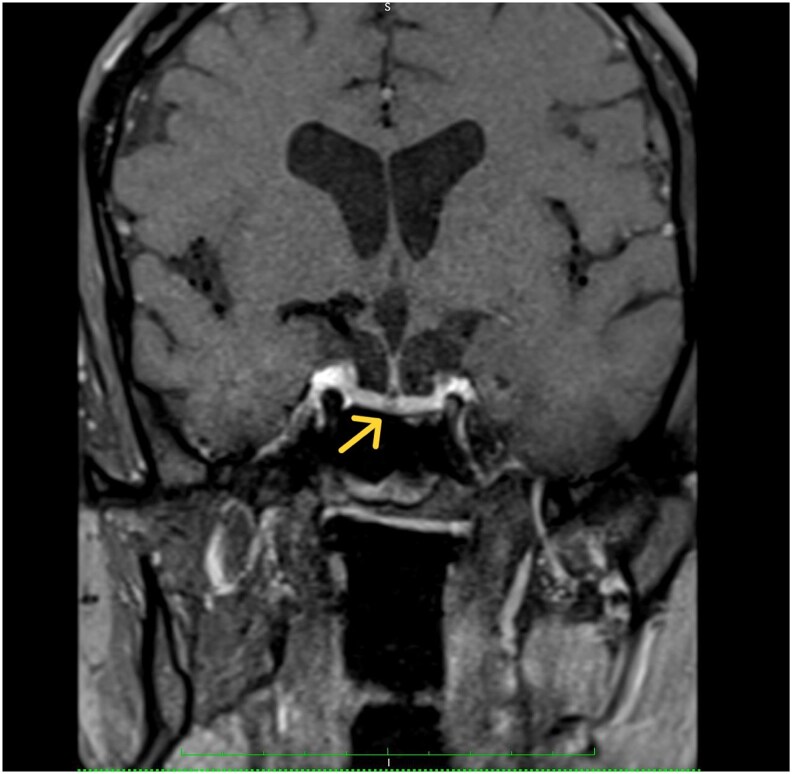
Magnetic resonance imaging of the pituitary showing a nonenhancing microadenoma.

Magnetic resonance imaging of the pituitary showed a 2.2-mm nonenhancing lesion within the pituitary, radiologically consistent with a microadenoma. There was no mass effect, stalk deviation, or optic chiasm compression (Fig. 5). In the absence of biochemical evidence of GH hypersecretion, this lesion was considered a nonfunctioning incidental microadenoma. Additional laboratory results are mentioned in Table 1. Mild hyperprolactinemia (24.1 ng/mL [SI: 1024 mIU/L]) was observed without radiologic evidence of stalk compression and was considered likely physiologic or stress-related. The other pituitary axes were fairly normal.

**Table 1:** Hormonal profile of the patient. Reference ranges correspond to the performing laboratory and assay used at the time of testing

Investigation	Patient value	Reference range
TSH	2.15 mIU/mL	0.4-4.5 mIU/mL
Total T4	8.88 µg/dL (114.3 nmol/L)	5.0-12.0 µg/dL (64.4-154.8 nmol/L)
Free T4	1.16 ng/dL (15 pmol/L)	0.8-1.8 ng/dL (10-23 pmol/L)
FSH	18.76 mIU/mL	1.5-12.4 mIU/mL
Prolactin	24.1 ng/mL (1024 mIU/L)	2-18 ng/mL (85-765 mIU/L)
Cortisol (Am sample)	8.5 µg/dL (235 nmol/L)	7-25 µg/dL (193-690 nmol/L)
IGF-1 (baseline)	75.9 ng/mL (9.95 nmol/L)	94-284 ng/mL (12.3-37.2 nmol/L), age-dependent
GH (post–OGTT suppression)	0.49 ng/mL (0.49 µg/L)	<1.0 ng/mL (<1 µg/L) after OGTT

Abbreviations: FSH, follicle-stimulating hormone; GH, growth hormone; IGF-1, insulin-like growth factor 1; OGTT, oral glucose tolerance test; T4, thyroxine; TSH, thyrotropin.

Given the combination of tall stature, supernumerary nipples, dysmorphic features, and intellectual disability, a syndromic overgrowth disorder was suspected.

Exome sequencing revealed a hemizygous nonsense pathogenic variant in *GPC3* (c.1159C > T; p.Arg387Ter). This variant introduces a premature stop codon, predicted to cause nonsense-mediated messenger RNA decay and loss of glypican-3 function [3]. The variant was heterozygous in the mother and hemizygous in the brother.

The variant has been reported in variant databases as pathogenic and is consistent with a loss-of-function mechanism underlying SGBS type 1.

## Treatment

No endocrine therapy was required as acromegaly was excluded. Pulmonary tuberculosis and bronchiectasis were managed by the pulmonology team. Mixed hearing loss was evaluated and hearing rehabilitation was advised. Genetic counseling was provided to the patient and family. Tumor surveillance with abdominal ultrasonography and serum α-fetoprotein (AFP) measurement was initiated.

## Outcome and follow-up

Cardiac evaluation and abdominal ultrasonography showed no organomegaly, structural cardiac abnormalities, or genitourinary anomalies. AFP levels were normal.

The patient was advised periodic follow-up for tumor surveillance and audiologic monitoring. Family members were counseled regarding X-linked inheritance and offered genetic testing.

## Discussion

This case underscores the importance of phenotype-driven diagnosis in patients presenting with acromegaloid facies. Although the initial clinical impression was acromegaly, the broader constellation of findings, like tall stature since childhood, supernumerary nipples, craniofacial dysmorphism, skeletal anomalies, and intellectual disability, strongly suggested a syndromic overgrowth disorder. In retrospect, the diagnosis of SGBS was clinically apparent before endocrine testing. Biochemical evaluation served appropriately to exclude GH.

Somatic overgrowth in SGBS reflects dysregulation of developmental growth signaling rather than endocrine hypersecretion. The *GPC3* gene encodes glypican-3, a glycosylphosphatidylinositol-anchored heparan sulfate proteoglycan expressed on the cell surface. Glypican-3 modulates multiple morphogen pathways, including Wnt, Hedgehog, bone morphogenetic protein, and fibroblast growth factor signaling. Loss of glypican-3 function disrupts spatial growth regulation during embryogenesis, leading to generalized overgrowth, visceromegaly, and skeletal disproportion [4-6]. Unlike acromegaly, which is mediated by excess circulating GH and IGF-1, SGBS represents a developmental overgrowth syndrome with normal GH axis physiology.

The differential diagnosis of disproportionate overgrowth in adults includes several genetic and endocrine conditions. X-LAG is characterized by early-onset pituitary hyperplasia or adenoma with markedly elevated GH and IGF-1 levels [7]. Sotos syndrome (NSD1-related) presents with advanced bone age, macrocephaly, and characteristic dolichocephalic facies but lacks supernumerary nipples [8]. Weaver syndrome (EZH2-related) includes camptodactyly and widened distal femora [9]. Beckwith-Wiedemann syndrome often demonstrates lateralized overgrowth and neonatal hyperinsulinism [10]. In contrast, the presence of supernumerary nipples, X-linked inheritance, and distinct craniofacial morphology is highly suggestive of SGBS. Recognition of these distinguishing features is critical to avoid unnecessary endocrine investigations and to ensure appropriate genetic evaluation.

The endocrine profile in this patient further reinforces the distinction between syndromic overgrowth and acromegaly. The serum IGF-1 concentration was below the age-adjusted reference range, and GH was appropriately suppressed during OGTT. These findings exclude acromegaly.

Hypoglycemia has been reported in SGBS, particularly in infancy. The proposed mechanism involves pancreatic islet cell hyperplasia and relative hyperinsulinism, paralleling other overgrowth syndromes [11]. Our patient did not report documented neonatal hypoglycemia, although reliable birth records were unavailable due to home delivery. The absence of documented neonatal complications does not exclude prenatal overgrowth, which may have been present based on maternal recall.

Tumor predisposition is an important component of SGBS management. Recent data estimate a tumor risk of 5.1%, most commonly Wilms tumor and hepatoblastoma. The risk appears highest in early childhood, but ongoing surveillance strategies are recommended [12]. In this patient, abdominal ultrasonography and AFP levels were normal at evaluation. Although adult tumor risk is less clearly defined, genetic confirmation allows structured surveillance and informed family counseling.

The family evaluation in this case further supports the X-linked inheritance pattern of SGBS. The hemizygous pathogenic variant in *GPC3* was identified in both affected brothers and in a heterozygous state in the mother. The brother exhibited milder phenotypic expression, illustrating intrafamilial variability. Such variability may reflect modifier genes, environmental factors, or stochastic developmental influences rather than differences in GH axis function [12].

This case highlights a key clinical lesson: when acromegaloid features are accompanied by congenital anomalies, supernumerary nipples, and neurodevelopmental delay, clinicians should prioritize syndromic evaluation. Endocrine testing should be used to exclude GH excess, but the diagnosis should be grounded in careful physical examination and pattern recognition. Early identification of SGBS enables appropriate tumor surveillance, multidisciplinary management, and genetic counseling.

## Learning points

- SGBS is a rare, X-linked overgrowth disorder that can clinically mimic acromegaly due to shared acromegaloid features such as macroglossia and coarse facies.
- Normal IGF-1 levels and adequate GH suppression should prompt consideration of genetic overgrowth syndromes when acromegaly is clinically suspected but biochemically excluded.
- Supernumerary nipples, developmental delay, and dysmorphic skeletal features serve as useful clinical clues differentiating SGBS from acromegaly and other causes of somatic overgrowth.
- Genetic testing for *GPC*3 variants is confirmatory and essential for diagnosis, allowing appropriate family screening and genetic counseling.
- Early recognition of SGBS enables tumor surveillance for Wilms tumor and hepatoblastoma and facilitates multidisciplinary care for associated systemic anomalies.

## Contributors

R.K.R., S.G., H.A., K.D., and G.H.K. contributed to the clinical evaluation, diagnosis, and management of the patient and to the acquisition and interpretation of clinical data. R.K.R., H.A., and V.B. contributed to the conception and design of the report and to data analysis and interpretation. R.K.R. and H.A. drafted the manuscript, and all authors critically revised it for important intellectual content. All authors approved the final version of the manuscript and agree to be accountable for all aspects of the work.

## Data Availability

Original data generated and analyzed during this study are included in this published article.

## Abbreviations

AFP: α-fetoprotein
GH: growth hormone
IGF-1: insulin-like growth factor 1
OGTT: oral glucose tolerance test
SGBS: Simpson-Golabi-Behmel syndrome
X-LAG: X-linked acrogigantism
